# DNA immunization with *in silico* predicted T-cell epitopes protects against lethal SARS-CoV-2 infection in K18-hACE2 mice

**DOI:** 10.3389/fimmu.2023.1166546

**Published:** 2023-04-11

**Authors:** Gry Persson, Katherine H. Restori, Julie Hincheli Emdrup, Sophie Schussek, Michael Schantz Klausen, McKayla J. Nicol, Bhuvana Katkere, Birgitte Rønø, Girish Kirimanjeswara, Anders Bundgaard Sørensen

**Affiliations:** ^1^ Evaxion Biotech A/S, Hoersholm, Denmark; ^2^ Department of Veterinary and Biomedical Sciences, Pennsylvania State University, University Park, PA, United States

**Keywords:** COVID-19, machine learning, T-cell vaccine, SARS-CoV-2, viral challenge model

## Abstract

The global SARS-CoV-2 pandemic caused significant social and economic disruption worldwide, despite highly effective vaccines being developed at an unprecedented speed. Because the first licensed vaccines target only single B-cell antigens, antigenic drift could lead to loss of efficacy against emerging SARS-CoV-2 variants. Improving B-cell vaccines by including multiple T-cell epitopes could solve this problem. Here, we show that *in silico* predicted MHC class I/II ligands induce robust T-cell responses and protect against severe disease in genetically modified K18-hACE2/BL6 mice susceptible to SARS-CoV-2 infection.

## 1 Introduction

SARS-CoV-2 and its related syndrome, COVID-19, have been associated with more than 6.8 million deaths worldwide and imposed economic burdens across the globe during the pandemic (1). First-generation vaccines against SARS-CoV-2 (mRNA-1273, BNT162b2, Ad26.CoV2, and NVX-CoV2373) were developed and approved with unprecedented speed to curtail the pandemic (2–5).

Although effective, these vaccines all rely on the same single antigen, the spike (S) protein (6, 7), and are designed to elicit the production of neutralizing antibodies against this target from the original SARS-CoV-2 strain (6, 7). Several publications highlight the importance, in addition to the induction of neutralizing antibodies (8, 9), of a strong cellular response to these vaccines in the control of SARS-CoV-2 infection (10–12). The shortcoming of such a strategy is the potential for the virus to evade immune cell detection, particularly through amino acid changes in the receptor-binding domain (RBD) of the S protein, and the emergence of new viral variants of concern (VOCs) (P. 13, 14; Q. 15). This, combined with waning vaccine-induced antibody levels over time (16–18), means that SARS-CoV-2 VOCs and other zoonotic novel coronaviruses continue to pose a threat (4, 19). Consequently, first-generation vaccines need to be regularly updated to efficiently target the altered antigens of novel variants (20, 21).

The global spread of SARS-CoV-2 has resulted in approximately 10% of the world’s population being infected (1). It has been reported that immune responses in previously infected subjects are stronger (humoral responses are more durable, cross-strain reactivity is greater, and T-cell epitope coverage is broader) than those observed in response to S protein vaccination. This provides evidence that it is possible to develop more robust vaccines that possess broader T-cell epitope coverage (10–12). Such strategies have been pursued recently, focusing on T-cell epitopes derived from structural proteins, with good immunological results. However, to our knowledge, no such vaccines have been evaluated in a mouse SARS-CoV-2 live viral challenge model (22, 23).

To generate a vaccine with the potential to generate broader protection and with less likelihood of being subject to antigenic drift, we designed and preclinically tested a T-cell vaccine composed of fully *in silico* predicted epitopes sampled from the entire SARS-CoV-2 genome using the RAVEN™ (Rapidly Adaptive Viral rEspoNse) computational platform. In summary, we show that such a vaccine, delivered as a plasmid DNA (pDNA) vaccine, induces reactive T cells to respond to 15 out of the 17 included epitopes in immunized C57BL/6J mice. Furthermore, we show that immunization of K18-hACE-2 x C57BL/6J mice with the T-cell pDNA vaccine protects against lethal disease upon live viral challenge with SARS-CoV-2, even in the absence of neutralizing antibodies.

## 2 Methods

### 2.1 Ethics statement

Immunogenicity studies in mice were performed at Evaxion Biotech A/S (Denmark) under license 2017-15–0201-01209, in accordance with 2010/63/EU. Live viral challenge studies in mice were performed at the Eva J. Pell Laboratory for Advanced Biological Research at the Pennsylvania State University (USA). All animal studies were performed in compliance with Protocol #01552 of the Institutional Animal Care and Use Committee of the Pennsylvania State University.

### 2.2 Design and production of the SARS-CoV-2 T-cell epitope pDNA vaccine

The T-cell epitope pDNA vaccine was designed by applying the RAVEN platform to the proteome of the SARS-CoV-2 isolate Wuhan-Hu-1 (accession no. NC_045512.2), as outlined below. Coding sequences from full-length SARS-CoV-2 proteins were used for deep learning-based MHC class I (tiling to 8-, 9, 10, and 11-mers) and class II ligand (15-mers) prediction using H-2-Kb and H-2-Db and the H-2-Iab alleles, respectively, with tools developed in house. The design of the final T-cell vaccine was achieved using a genetic algorithm that generated 10,000 constructs by randomly combining selected MHC class I and II ligand-rich regions (“hotspots”) from across the entire SARS-CoV-2 genome, applying the following constraints: (1) constructs were 1,440 bp in length; (2) constructs were selected from different viral genes; and (3) MHC class I ligands were selected in a 2:1 ratio. The highest-scoring 20% of designs were then supplemented with 5% newly generated random constructs, and this pool was used to combine two randomly selected constructs until 10,000 constructs were again reached in the pool. This process was repeated 200 times to create a highly optimized set of constructs with strong MHC binders meeting the stated requirements. The final selected hotspots were joined by GSGSGSGSGS linkers, and codon-optimized (mouse) DNA inserts were synthesized (Aldevron, USA), and cloned in a pTVG4 DNA plasmid (5′ *Not*I: GCGGCCGC and 3′ *Bam*HI: GGATCC) encoding mouse CCL19 as an antigen-presenting cell targeting (APCt) unit and a human IgG3 heavy chain dimerization unit (Aldevron, USA) to create the final pDNA vaccine (APCt-CoV-T). Empty pTVG4 DNA vector containing no T-cell antigens (APCt-Mock) were used as controls. An overview of the T-cell hotspots and predictions, encoded in the APCt-DNA T-cell vaccine, can be found in **Table S1** and **Figures S1** and **S2**.

### 2.3 Mouse strains

Immunogenicity studies were performed in 5–6 weeks-old female C57BL/6J mice (Janvier Laboratories, France). K18-B6.Cg-Tg(K18-ACE2)2Prlmn/J mice (K18-hACE2 Jackson Laboratories, ME, USA) were used for the SARS-CoV-2 challenge study. The K18-hACE2 mice were backcrossed to C57BL/6J mice (Jackson Laboratories) for seven generations to generate K18-hACE2 mice on a C57BL/6J background. The C57BL/6J H-2Kb haplotype of the K-18-hACE2/BL6 mice was verified by haplotyping using PCR and flow cytometry (**Tables S2**, **S3**, data not shown). Immunogenicity and challenge studies were performed in female and male K18-hACE2/BL6 mice at 8–10 weeks of age.

### 2.4 Immunogenicity evaluation in C57BL/6J mice

Mice were immunized with a total dose of 25 μg pDNA/100 μL (APCt-CoV-T and APCt-Mock) in 1× phosphate-buffered saline (PBS) formulated 1:1 with 6% block co-polymer poloxamer 188 (kindly provided by BASF, Germany). The mice received intramuscular (i.m.) injections of 50 μL into the tibialis anterior muscles of each hind leg once a week for 5 weeks for all studies except for tissue sampling studies, in which case the mice received two immunizations 4 weeks apart. Sera were isolated from tail vein blood collected throughout the study and at study termination 2 or 4 weeks after the last immunization. At termination, spleens were collected, and single-cell suspensions were prepared in accordance with the manufacturer’s instructions using a gentleMACS dissociator (Miltenyi Biotec, Germany).

### 2.5 SARS-CoV-2 viral stock preparation and storage

SARS-CoV-2 isolate USA-WA1/2020 was received from BEI Resources, the National Institute of Allergy and Infectious Diseases (NIAID), and the National Institutes of Health (NIH) (#NR-5228). Stock preparation and storage were performed as previously described (24). The titer of the propagated virus stock used for challenge and in mouse lung tissue after challenge infection was determined as previously described (24, 25). Lung samples were collected in 0.5 mL of Dulbecco’s modified Eagle medium (DMEM) and homogenized using a Qiagen TissueLyser II.

### 2.6 SARS-CoV-2 challenge studies in K18-hACE2/BL6 mice

Groups of eight K18-hACE2/BL6 mice were immunized five times as described above. Four weeks after the last immunization, the mice were moved to an Animal Biosafety Level 3 facility for an acclimation period of 5 days before the challenge. Inoculum for the challenge infection was prepared from frozen viral stocks by diluting the virus with PBS to the appropriate concentration. The mice were challenged by an intranasal (i.n.) instillation of 1,000 × TCID_50_ (the median tissue culture infectious dose) of SARS-CoV-2 (USA-WA1/2020) under anesthesia using isoflurane. Survival, body weight, and clinical symptoms (**Table S4**) following the challenge were monitored daily for 14 consecutive days post-challenge. At termination, lungs were harvested from a subgroup of the mice, while viral load determination using the TCID_50_ assay in Vero6 cells, as described above, was performed on lung homogenate from the remaining lungs. Naive mice (i.e., those not vaccinated and not challenged) were included in all the studies as healthy control animals.

### 2.7 Evaluation of T-cell responses using IFN-γ enzyme-linked immunosorbent spot assay

Hotspot amino acid sequences were synthesized as lyophilized peptides (Pepscan, the Netherlands), dissolved in dimethyl sulfoxide (DMSO) (Merck, #D8418) to a concentration of 10 mg/mL, and used in *ex vivo* immune assays (**Table S1**). An enzyme-linked immunosorbent spot (ELISpot) assay was performed in accordance with the manufacturer’s protocol (BD Biosciences) with minor adjustments. Each well was then seeded with 5 ×10^5^ murine splenocytes and stimulated for 18–20 hours with individual peptides or peptide pools (5 μg/mL of each peptide) along with a positive control (concanavalin A, 5 µg/well) and a negative control (culture medium containing <0.6% DMSO). The experiments were run in duplicate. Spot-forming units (SFUs) were counted using an ImmunoSpot analyzer and software (Cellular Technology Limited). A positive ELISpot response was defined as >3 times the standard deviation of the negative control or >20 SFUs per 1 ×10^6^ cells.

### 2.8 Automated immunohistochemistry

Lung samples from three APCt-CoV-2-T-vaccinated mice, the only surviving APCt-mock mouse, and two naive mice were fixed in 10% neutral-buffered formalin and stored in 70% ethanol until analysis. Samples were fully dehydrated, embedded in paraffin, and sectioned prior to automated staining of CD45 using a Ventana Discovery Ultra instrument (Roche). Briefly, 5-μm-thick paraffin sections were processed for epitope demasking using a CC2 buffer (Roche) at 97°C for 16 minutes. Slides were incubated with a rabbit anti-mouse CD45 antibody (diluted at 1:2000) (**Table S3** for details) and the CD45 protein was detected using horseradish peroxidase (HRP)-conjugated anti-rabbit antibody (omniMap-Rabbit-HRP, Roche). Images were acquired from digital slides obtained using a Zeiss AxioScan equipped with a 20× objective.

### 2.9 Data analysis

All analyses were performed using GraphPad Prism 9 for Mac OS X. Analysis of immunohistochemistry (IHC) images was carried out using QuPath version 0.3.2 (26).

## 3 Results

**Figure 1:**
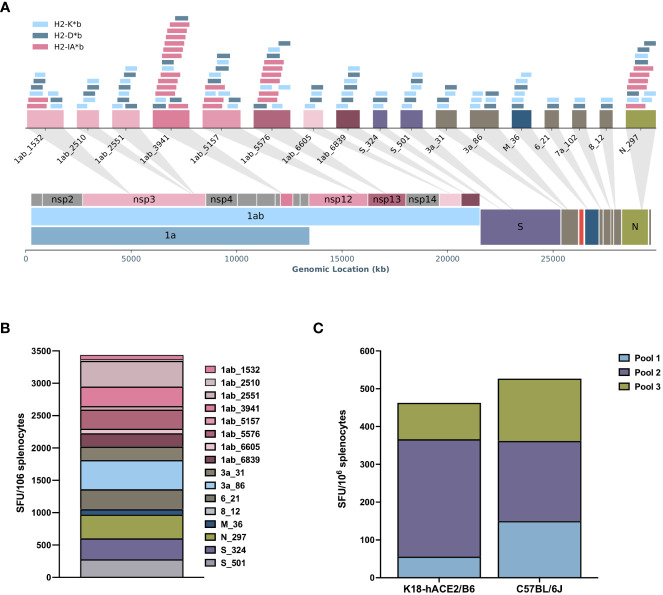
The RAVEN™ (Rapidly Adaptive Viral rEspoNse) antigen-presenting cell targeting (APCt)-CoV2-T vaccine induces specific T-cell responses in C57BL/6J and K18-hACE2/BL6 mice. **(A)** Schematic overview of the in silico RAVEN predicted T-cell hotspots encoded in the APCt-CoV2-T-cell vaccine. A T-cell hotspot represents a region with several predicted major histocompatibility complex (MHC) class I (H2-Kb, H2-Db) and MHC class II (H2-Iab) binders. **(B)** IFN-γ enzyme-linked immunosorbent spot (ELISpot) of splenocytes from APCt-CoV2-T-vaccinated C57BL/6J mice (*n* = 4) shows a response to 15 of the 17 peptide hotspots included. **(C)** IFN-γ ELISpot using peptide pools for stimulation of splenocytes from C57BL/6J (*n* = 3) and K18-hACE2/BL6 (*n* = 4) mice show a comparable response to the APCt-CoV2-T vaccine. No response was noted for APCt-mock-vaccinated animals (data not shown).

To evaluate the immunogenic potential of *in silico* predicted MHC class I and class II ligands from the entire SARS-CoV-2 genome (**Figure 1A**), we designed a polyepitope construct using an evolutionary algorithm, resulting in a design with 17 vaccine-encoded T-cell hotspots, each representing a region with multiple predicted MHC class I (H2-Kb, H2-Db) and MHC class II (H2-IAb) binders (**Figure 1A**). The final construct was then cloned in a DNA plasmid for fusion with a chemokine (CCL19), allowing for the targeting and recruitment of antigen-presenting cells to generate the APCt-CoV2-T vaccine (**Figure 1A**, **Table S1**, **Figure S1**). *In vivo* testing showed that APCt-CoV2-T-vaccinated (two immunizations) C57BL/6J mice mounted a broad and strong cellular response, as evidenced by T-cell reactivity upon peptide restimulation toward 15 of the 17 (87.5%) vaccine-encoded hotspots (**Figure 1B**). The IFN-γ T-cell response for peptide 8_12 did not meet the detection criterion, and peptide 7a_102 was not included in the analysis owing to failed peptide synthesis. The IFN-γ T cells in the K18-hACE/BL6 knock-in mice (five immunizations) showed a similar response when stimulated with pools of vaccine-encoded peptide epitopes (**Figure 1C**). Intracellular cytokine staining of stimulated splenocytes from APCt-CoV2-T-vaccinated C57BL/6J mice demonstrated the induction of polyfunctional CD4^+^ and CD8^+^ T cells positive for TNF-α and IFN-γ (**Figure S3**). No T-cell response was noted in APCt-mock-vaccinated mice (data not shown).

**Figure 2:**
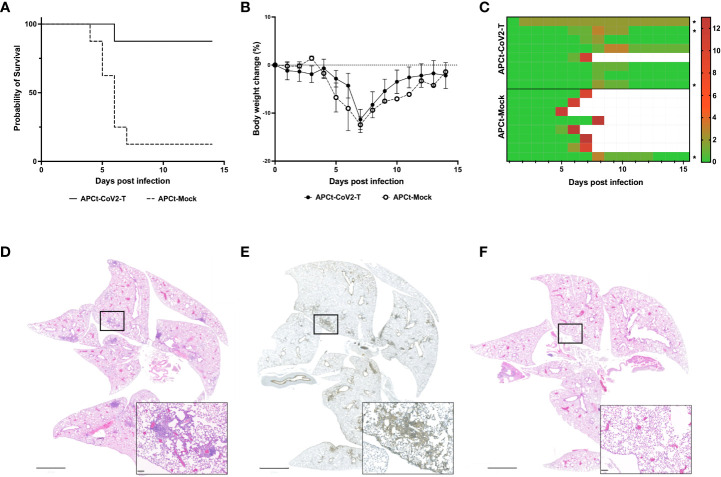
The RAVEN™ (Rapidly Adaptive Viral predicted rEspoNse) antigen-presenting cell targeting (APCt)-CoV2-T vaccine increases survival in SARS-CoV-2-infected K18-hACE2/BL6 mice. **(A)** Survival plot, **(B)** Body weight change, and **(C)** the total sum of clinical scores. The mice were monitored for 14 consecutive days post-infection. **(D)** Hematoxylin and eosin **(H&E)** staining and **(E)** CD45 immunohistochemistry (IHC) staining of the lungs of APCt-CoV2-T vaccinated mice. **(F)** H&E staining of the lungs from naïve mice at day 14 post-infection. The IHC images are representative of the three lungs evaluated (**Figure S5**). Scale bars are 2 mm and 100 μm. *Mice in which H&E and IHC staining of lung tissue was performed.

To evaluate the functionality of the induced T cells, K18-hACE2/BL6 mice were immunized once a week for 5 weeks to ensure sufficient T-cell responses to the APCt-CoV2-T vaccine and subsequently challenged i.n. with a lethal dose of SARS-CoV-2 (USA-WA1/2020). The APCt-CoV2-T-vaccinated mice had a survival rate of 87.5% (7/8), whereas only one in eight (12.5%) of the APCt-Mock-vaccinated mice survived the viral infection (**Figure 2A**). The APCt-CoV2-T- and APCt-Mock-vaccinated mice exhibited various clinical signs, mainly body weight loss and ocular infection (**Figures 2B, C**, **S4**). APCt-Mock-vaccinated mice presented with more severe clinical symptoms throughout the challenge study. Anti-RBD IgG antibodies were not induced by the APCt-CoV2-T vaccine (**Figure S5**); therefore, the protection against lethal challenge is solely T-cell mediated.

All surviving APCt-CoV2-T-vaccinated mice had cleared the infection by day 14 post-challenge, with no detectable signs of the virus in lung homogenates (data not shown). Hematoxylin and eosin (H&E) and IHC staining of lung sections from APCt-CoV2-T- and APCt-mock-vaccinated mice was therefore performed to confirm infection post-challenge and the expected focal tissue remodeling with immune cell filtration in multiple tertiary lymphoid structures was observed. The cells in lymphoid structures stained positive for CD45^+^, indicating immune cell infiltration (**Figures 2D, E**). No such staining of lymphoid structures was seen in the lungs of naïve mice (**Figure 2F**).

Taken together, these findings verify the ability of *in silico* tools (i.e., RAVEN) to identify and select relevant functional immunogenic T-cell regions in the SARS-CoV-2 genome that can protect against lethal COVID-19.

## 4 Discussion

The majority of the approved COVID-19 vaccines target the spike protein or a subunit thereof and induce antibody-mediated protective immunity. Here we demonstrate the feasibility of using novel machine learning tools to design a purely T-cell epitope-based vaccine (APCt-CoV2-T) that elicits abroad immunogenic response to 15 of 17 included genomic hotspots containing MHC ligands from the SARS-CoV-2 genome, including structural proteins, accessory factors, and non-structural proteins. This novel *in silico* predicted and designed T-cell vaccine delivered with an APCt chemokine (CCL19), further induced the production of multifunctional (TNF-α^+^/IFN-γ^+^) CD4^+^ and CD8^+^ T cells in vaccinated mice, correlating well with our predictions, as depicted in **Figures 1A** and **S2**. Unfortunately, it was not possible to carry out any correlation analyses in the current study between viral load and the protective T-cell response, as all mice had cleared the infection at day 14 post-infection. Type II interferons, such as IFN-γ, are synthesized mainly by activated natural killer (NK) cells and activated T cells in response to virus infection and are central to regulating an anti-viral immune response (27). Importantly, the APCt-CoV2-T vaccine protected against an *in vivo* SARS-CoV-2 viral challenge in K18-hACE2/BL6 mice, demonstrating the induction of a favorable cellular immune response with anti-viral and immune-stimulating properties that inhibits viral replication. To our knowledge, this is the first study to show *in vivo* protection from severe COVID-19 by a fully *in silico* predicted and designed T-cell vaccine. Two additional coronaviruses have resulted in severe human disease [SARS-CoV-1 (28) and MERS-CoV (29)] in recent decades, while four other, less virulent, strains (OC43, HKU1, NL63, and 229E) account for 2%–14% of common cold cases (30). Pre-existing T-cell immunity has been detected in patients with no prior history of COVID-19, most probably because of previous exposure to one of the four common cold coronaviruses (31). Moreover, individuals who recovered from SARS-CoV-1 infection had SARS-CoV-2 cross-reactive CD4^+^ and CD8^+^ T cells 17 years after infection (31). These observations, combined with the presented findings, support the concept that a T-cell vaccine would be resistant to antigenic drift and therefore may promote durable antiviral memory responses in humans. Ongoing studies are focused on deconvoluting the variance and type of T-cell response against predicted epitopes for optimization of the prediction algorithms. The ability of the applied *in silico* design approach to successfully locate and combine relevant immunogenic “hotspots” from across a viral genome in a pathogen-agnostic manner paves the way for the potential rapid design of novel T-cell vaccines against emerging/seasonal viral diseases. In addition, such an approach may find a potential application in the clearance of chronic viral infections, owing to the broadness of the selected epitopes.

## Data availability statement

The raw data supporting the conclusions of this article will be made available by the authors, without undue reservation.

## Ethics statement

The animal study was reviewed and approved by the Eva J. Pell Laboratory for Advanced Biological Research at the Pennsylvania State University.

## Author contributions

GP, AS, and GK designed and supervised the studies. MK and AS developed the in silico platform RAVEN™ and designed the plasmid DNA vaccines. GP performed the C57BL/6J mouse immunization studies, while KR, BK, and MN performed the K18-hACE2/BL6 mouse immunogenicity and challenge studies. JE, SS, and GP evaluated *ex vivo* responses by ELISpot, intracellular cytokine staining (ICS), and enzyme-linked immunosorbent assay. The final analysis of all data was performed by GP and JE. GP, JE, AS, and KR drafted the manuscript, and BR and GK assisted with the final revision of the manuscript. All authors contributed to the article and approved the submitted version.
