# Supplementary material for: DNA immunization with in silico predicted T-cell epitopes protects against lethal SARS-CoV-2 infection in K18-hACE2 mice
Source: Front Immunol. 2023 Apr 11;14:1166546. doi: 10.3389/fimmu.2023.1166546 (PMC10126292; doi:10.3389/fimmu.2023.1166546)
Supplement: Supplementary file 1 [file DataSheet_1.pdf]

Supplementary Material

DNA immunization with *in silico* predicted T-cell epitopes protects against lethal SARS-CoV-2 infection in K18-hACE2 mice

Gry Persson, Katherine H. Restori, Julie Hincheli Emdrup, Sophie Schussek, Michael Schantz Klausen, McKayla J. Nicol, Bhuvana Katkere, Birgitte Rønø, Girish Kirimanjeswara, Anders Bundgaard Sørensen*

*** Correspondence:** Corresponding Author abs@evaxion-biotech.com

1 Supplementary Figures and Tables

Table S1. Overview of APCt-DNA-T cell vaccine encoded T-cell hotspots. Peptides were either used individually or as peptide pools in *ex vivo* stimulation of splenocytes for ELISPOT and ICS.

* Peptide ID 7a_102 failed synthesis and was not tested in ELISPOT and ICS

Gene	Peptide ID	Sequence (5'→3')	Peptide pool no.
Nsp3	1ab_1532	KSVYYTSNPTTFHLDGEVITFDNLKTLL	#1
Nsp3	1ab_2510	KTYERHSLSHFVNLDNL	
Nsp3	1ab_2551	SSAKSASVYYSQLMCQPILLL	
Nsp8	1ab_3941	QAIASEFSSLPSYAAFATAQEAYEQAVA	
Nsp12	1ab_5157	FNSTYASQGLVASIKNFKSVLYYQNNVFM	
Nsp13	1ab_5576	YPTLNISDEFSSNVANYQKVGMQKYSTL	
Nsp15	1ab_6605	SVGPKQASLNGVTLI	#2
Nsp16	1ab_6839	MNVAKYTQLCQYLNTLTL	
ORF3a	3a_31	TATIPIQASLPFGWLI	
ORF3a	3a_86	FVTVYSHLLLVAAGLEAPFLYL	
ORF6	6_21	FKVSIWNLDYI	
ORF8	8_12	VAAFHQECSL	#3
M	M_36	FAYANRNRFLYIIKL	
N	N_297	YKHWPQIAQFAPSASAFFGMSRI	
S	S_324	SIVRFPNITNL	
S	S_501	GVGYQPYRVVVLSFELL	
ORF7a*	7a_102	IVAAIVFITLC	N/A

Table S2. Primers used for genotyping and haplotyping.

Primer	Sequence (5’->3’)
Genotyping:[a]	
Transgene fwd (hKRT18flank)	GACCCCTGAGGGTTTCATATAG
Common rev (mChr2)	CACCAACACAGTTTCCCAAC
Wild type fwd (mChr2)	AAGTTGGAGAAGATGCTGAAAGA
Haplotyping:	
H-2Kb fwd	CAGTCAGCTCTTACCCCCATT
H-2Kb rev	CGTAGCCGACTTCCATGTAC
H-2Kd fwd	GTTCCAGCGGATGTTCGGC
H-2Kd rev	CGTCTCATTCCCGAGCTCCA
H2-Ab1 fwd	CCCTCAACCACCACAACACT
H2-Ab1 rev	ACATCTTGCTCCAGGCAGAC
H-2Ld fwd	CCCTGACCTGGCAGTTGAAT
H-2Ld rev	CACAAAAGCCACCACAGCTC

[a] PCR was performed according to Benavides, F. *et al.* PCR-based microsatellite analysis for differentiation and genetic monitoring of nine inbred SENCAR mouse strains. *Lab Anim* 35, 157–162 (2001), and genotype was confirmed by separated PCR Assay from The Jackson Laboratory (Protocol 38276).

Table S3. Flow cytometry antibody panels for haplotyping and intracellular cytokine staining (ICS) and antibodies for IHC. NR: Not relevant.

Antibody	Details	Provider	Cat.no.
Antibodies for Haplotyping:			
Fixable Viability Dye eFluor™ 780	-	eBioscience™	65-0865-14
B220-Pacific Blue	Clone: RA3-6B2	BioLegend®	103227
I-A(b)-PE	Tetramer: PVSKMRMATPLLMQA	NIH Tetramer Core Facility	35705
H-2K^b-PE	Tetramer: SIINFEKL	NIH Tetramer Core Facility	58560
CD3-PE/Cy7	Clone: KT3.1.1	BioLegend®	155621
CD4-Alexa Fluor®700	Clone: RM4-5	BioLegend®	100536
CD8-PerCpCy5.5	Clone: 53-6.7	BioLegend®	100734
CD90.1-AF488	Clone: OX-7	BioLegend®	®202506
CD90.2-BV511	Clone: 53-2.1	BioLegend®	140319
Antibodies for ICS:			
GloCell Fixable Viability Dye	NR	Stemcell	75010
CD3-FITC	Clone: 145-2C11	Biolegend®	100306
CD4-PE/Cy7	Clone: GK1.5	BD Biosciences	563933
CD8α-BV786	Clone: 53-6.7	BD Biosciences	563332
FCR block (anti-CD16/32)	Clone: 93	Biolegend®	101302
TNF-α-BV421	Clone: MP6-XT22	BD Biosciences	563387
IFN-γ BV650	Clone: XMG1.2	BD Biosciences	563854
Antibodies for IHC:			
CD3	Clone: SP162	AbCam	ab135372
CD45	Clone: RM1007	AbCam	ab281586

Table S4. Clinical scores and humane endpoints.

	**Severity level: 0**	**Severity level: 1**	**Severity level: 2**	**Severity level: 3**
**Activity level**	Alert	Reduced activity, but alert	Lethargic, moves slowly	Inactive, non-responsie
**Weight**	No weight loss	Minor weight loss (5-10%)	Lose <25% of initial BW	Lose >25% of initial BW
**Dehydration**	Normal (tented skin snaps back to normal quickly)	Mild dehydration (skin not pliable and returns to normal in about 30 s)	Mild dehydration (tented skin returns to normal >1 min)	Severe (skin over shoulders remains tented when scruffed)
**Breathing**	Normal	Rapid breathing	Labored breathing	Difficult breathing (gasping)
**Posture**	Normal	Hunched with rough coat (piloerection)		
**Eye**	Normal	Slight conjunctivitis (beginning, or one eye)	Conjunctivitis (in both eyes)	Severe conjunctivitis (eyes are crusty and opaque)

Clinical scoring parameters to measure pain and distress for removal criteria:

- If the mouse loses < 20% of bodyweight and has no symptoms of severity level 3, mice will be continued in the study and monitored once daily.
- If the mouse loses >20% of bodyweight, but still appears active (severity level 1), mice will be continued in the study and monitored once daily.
- If the mouse loses >20% of bodyweight but have moderate other symptoms (severity level 2), mice will be continued in the study and monitored twice daily.
- If the mouse loses >20% of bodyweight and shows any severe signs (severity level 3), mice will be removed from the study (euthanize by CO2 asphyxiation).
- If mice lose >25% bodyweight they will be immediately removed from the study (euthanized by CO2 asphyxiation).
- If the mouse has eye symptoms with no other changes, mice will be continued in the study and monitored once daily.

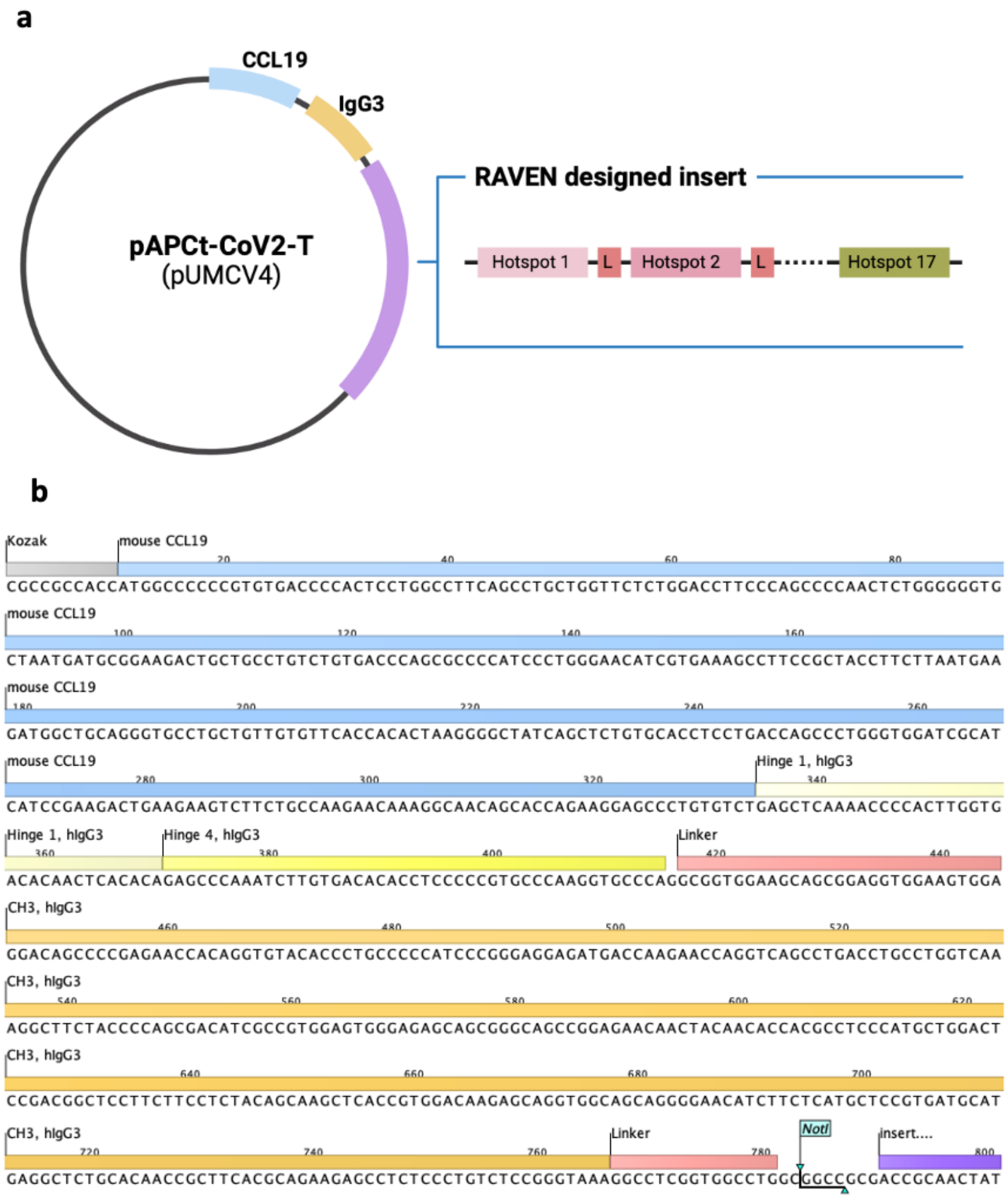

Figure S1. Schematic overview of the APCt-CoV2-T vaccine plasmid DNA. Codon-optimized DNA inserts encoding the RAVEN™ predicted hotspot sequences (cf. Table S1) tandem connected by glycine and serine (GSGSGSGSGS) linker elements were cloned in research-grade standard pTVG4 DNA plasmid (a) encoding mouse CCL19 as an antigen presenting cell targeting (APCt) unit and a human IgG3 heavy chain dimerization unit (b). The plasmid contains a CMV-driven expression casette and a kanamycin resistance gene for selection. The pTVG4 DNA plasmid was upscaled by Aldevron. Empty pTVG4 DNA vectors with no cloned RAVEN™ hotspot insert were used as controls.

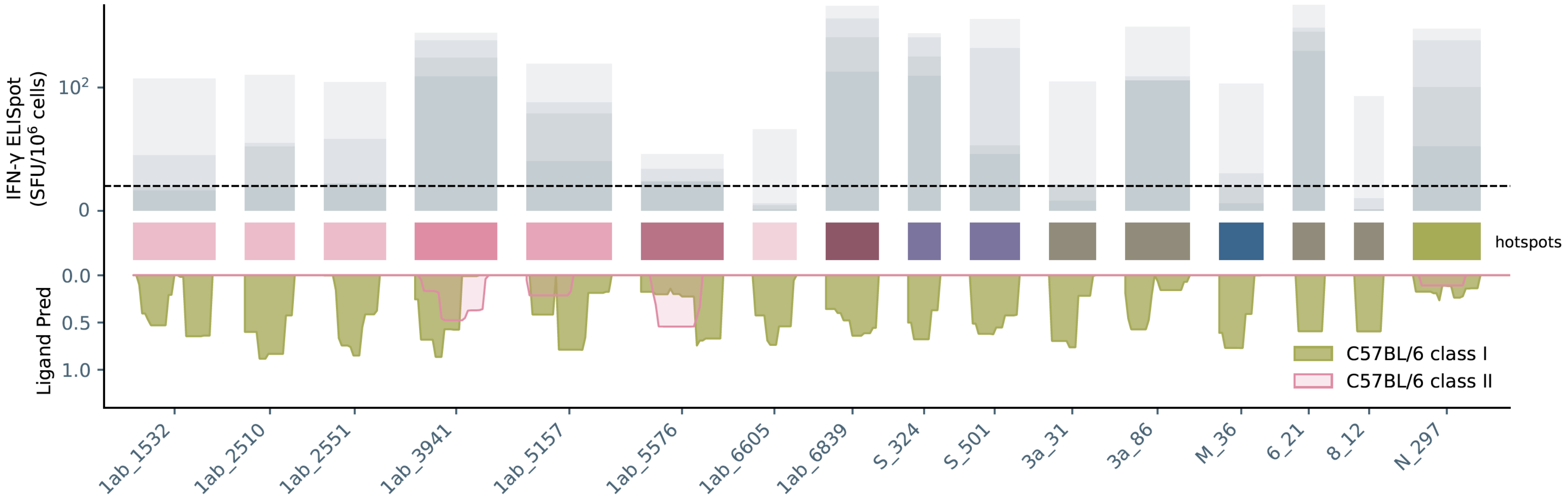

Figure S2. Mirror plot of RAVEN™ predictions and corresponding ELISpot read-outs. Predictions for C57BL/6 MHC class I (green) and MHC class II (light pink) is depicted under each hotspot. Each hotspot contains multiple predicted MHC class I/II ligands where the ligand prediction score ranges from 1 to 0, with 1 being highest predicted level and 0 is the lowest. IFN-γ ELISpot read-out shown above each hotspot as the number of spot forming units (SFU) per 10^6 cells from mice vaccinated twice with the APCt-CoV2-T vaccine plasmid. For the ELISpot, spleenocytes was from vaccinated mice was stimulated with peptides covering each of the entire hotspot regions. Shading in response indicate the variation in response between mice (N=4), and the dotted line indicate 25 SFU/10^6 cells as the lowest level of response.

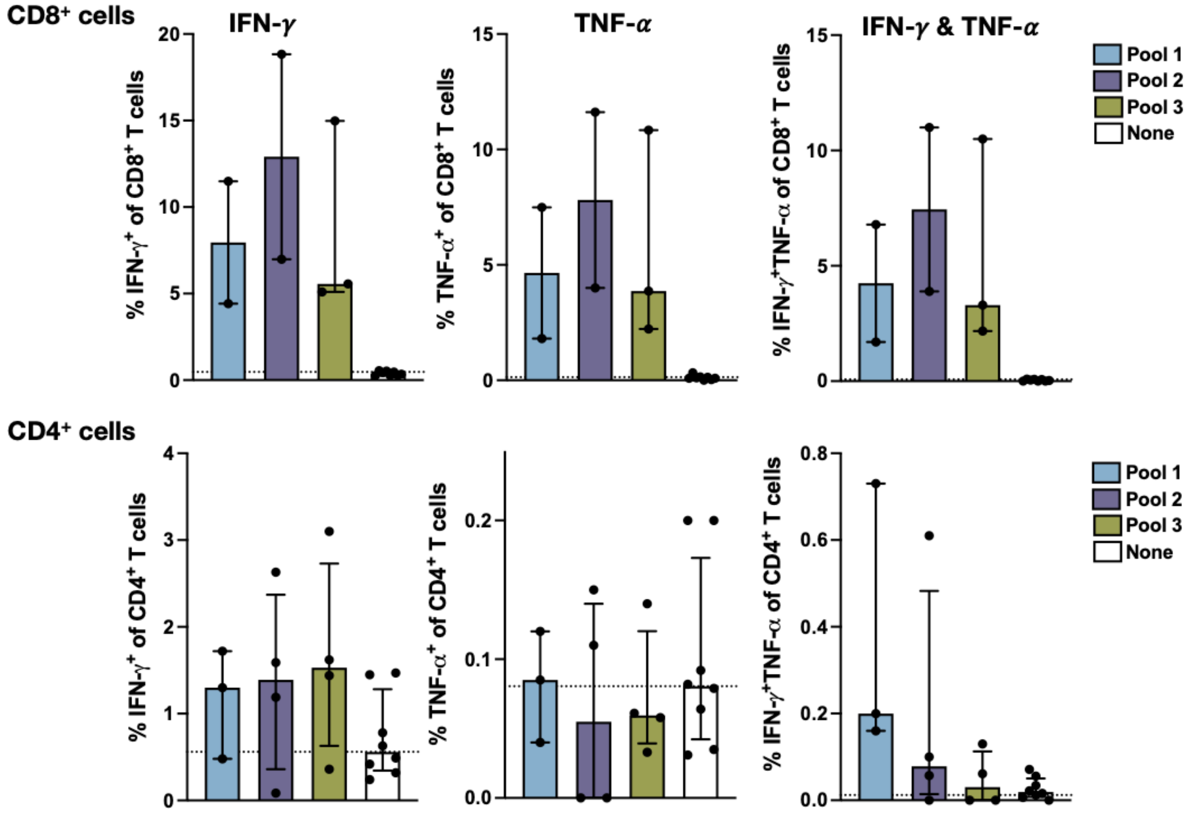

Figure S3. Intracellular cytokine staining (ICS) and flow cytometry of restimulated splenocytes from C57BL/6J vaccinated mice. Cryoperserved single-cell suspensions isolated from C57BL/6J murine spleens were thawed and washed in RPMI 1640 GlutaMax media (Gibco) containing 10% FBS (Gibco #10500-064). 1 x 10^6 live cells / 200 μL were stimulated with peptide pools (1 μg/mL of each peptide; Table S1), culture media alone (negative control) or with 0.4 μL leukocyte activation cocktail (LAC; BD Biosciences, #550583; not shown). Brefeldin A and Monensin (GolgiPlug™ and GolgiStop™; dilution of 1:1,000, BD Biosciences) were added to the cells after two hours of incubation at 37°C with 5% CO_2 and stained for flow cytometry analysis after 18-20 hours (cf. Table S3). For intracellular staining, fixation and permeabilization was done using 1X fixation buffer (eBioscience) and permeabilization buffer (eBioscience, 1:10), respectively. All staining were performed in PBS containing 2% FBS. Flow cytometry data were analyzed with FlowJo version 10.8.1 (Becton Dickinson). Each dot represents a pool of splenocytes from two individual C57BL/6J mouse ID's. T-cell reactivity was defined as the percentage of live $CD8^+$ or $CD4^+$ T-cells stained positive for at least one of the two markers (TNF-α and IFN-γ). Bars show median response with the dotted line representing the median background level of response in unstimulated cells.

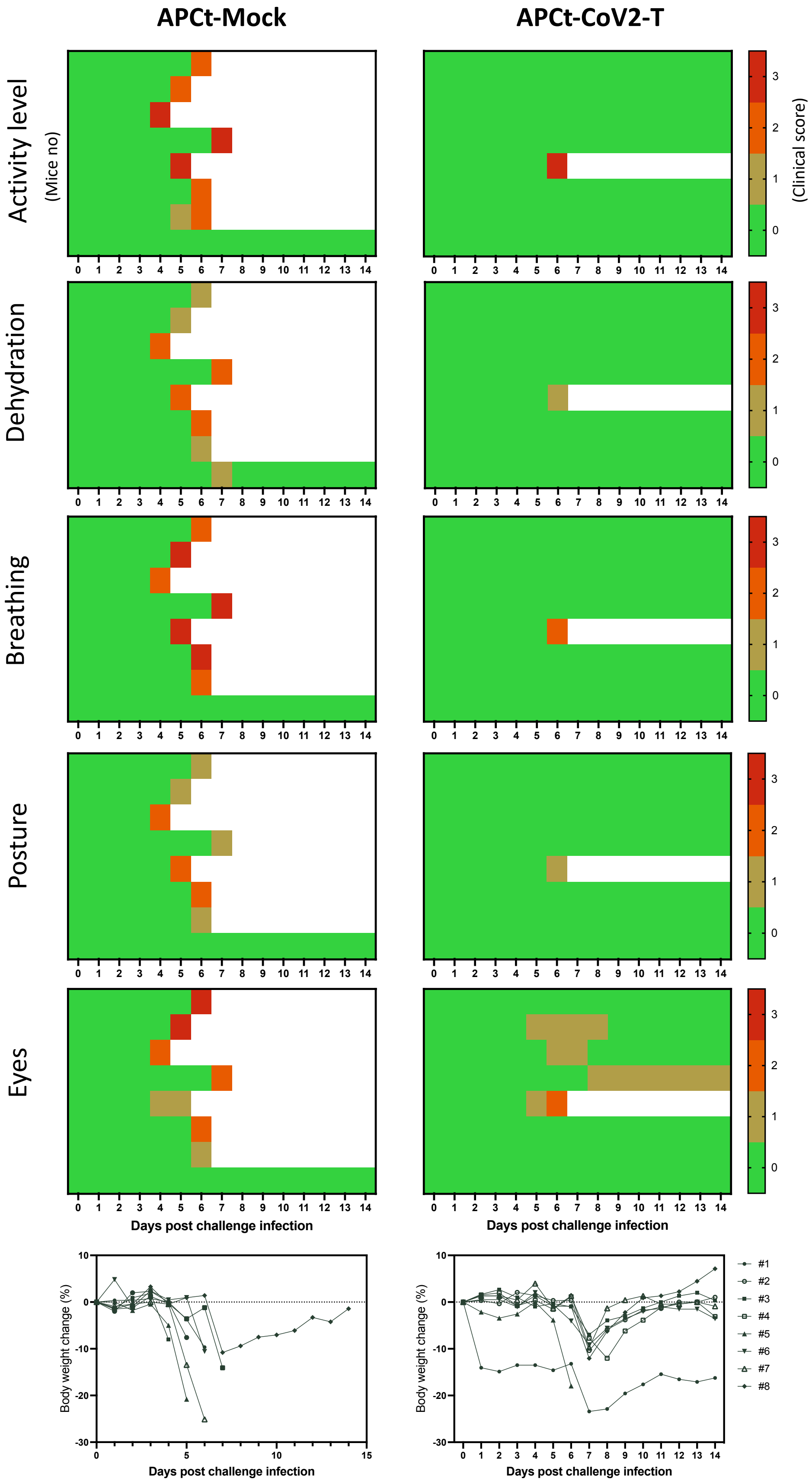

Figure S4. Clinical scores and body weight change for individual mice vaccinated with either APCt-Mock or APCt-CoV2-T vaccine (n=8 in each group). See Table S4 for details.

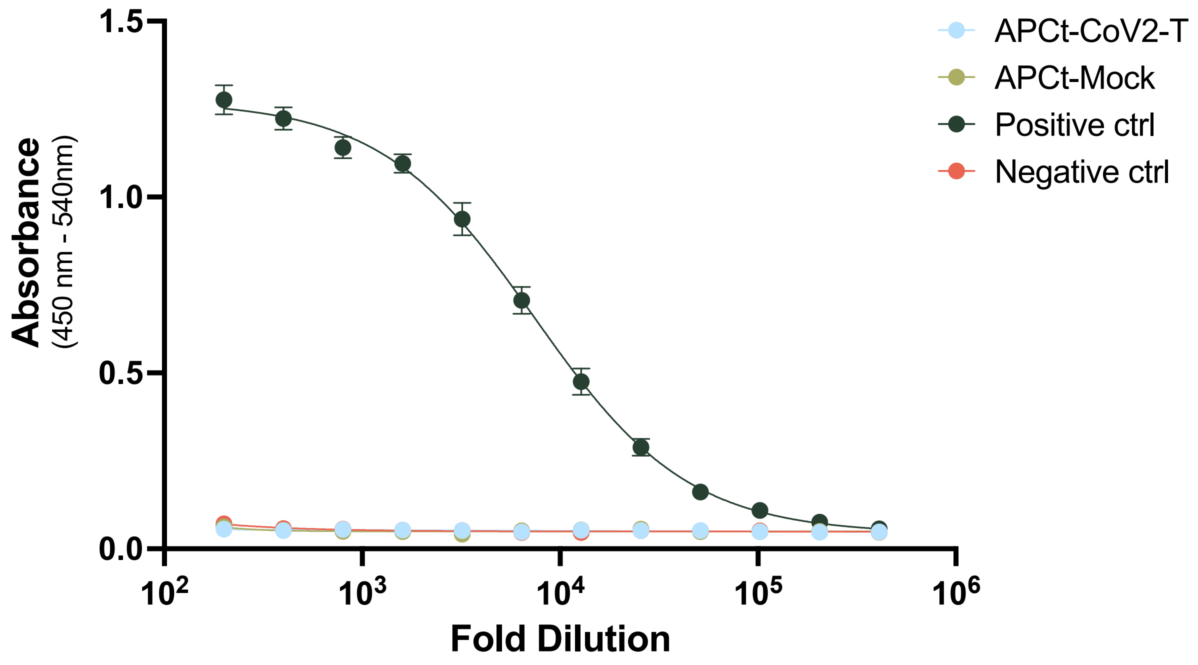

Figure S5. **Anti-RBD IgG ELISA**. ELISA was used to verify the absence of potential neutralizing antibodies. In short, ELISA, MaxiSorp microtiter plates (Thermo scientific were coated with 1 mg/mL SARS-CoV-2 recombinant RBD protein (Proteogenix). RBD-specific total IgG was detected by HRP-conjugated polyclonal rabbit anti-mouse IgG (Sigma-Aldrich) using 1-Step Slow TMB-ELISA (TMB Slow) (Thermo Fisher) for development. The end result is a number of absorbance values (450 nm minus 540 nm). Positive and negative ELISA controls consisted of sera from C57BL/6J mice s.c. vaccinated with 10 ug RBD protein vaccine formulated with 2% alumhydroxid as prime/boost (D0, 28) or alumhydroxid alone, respectively.

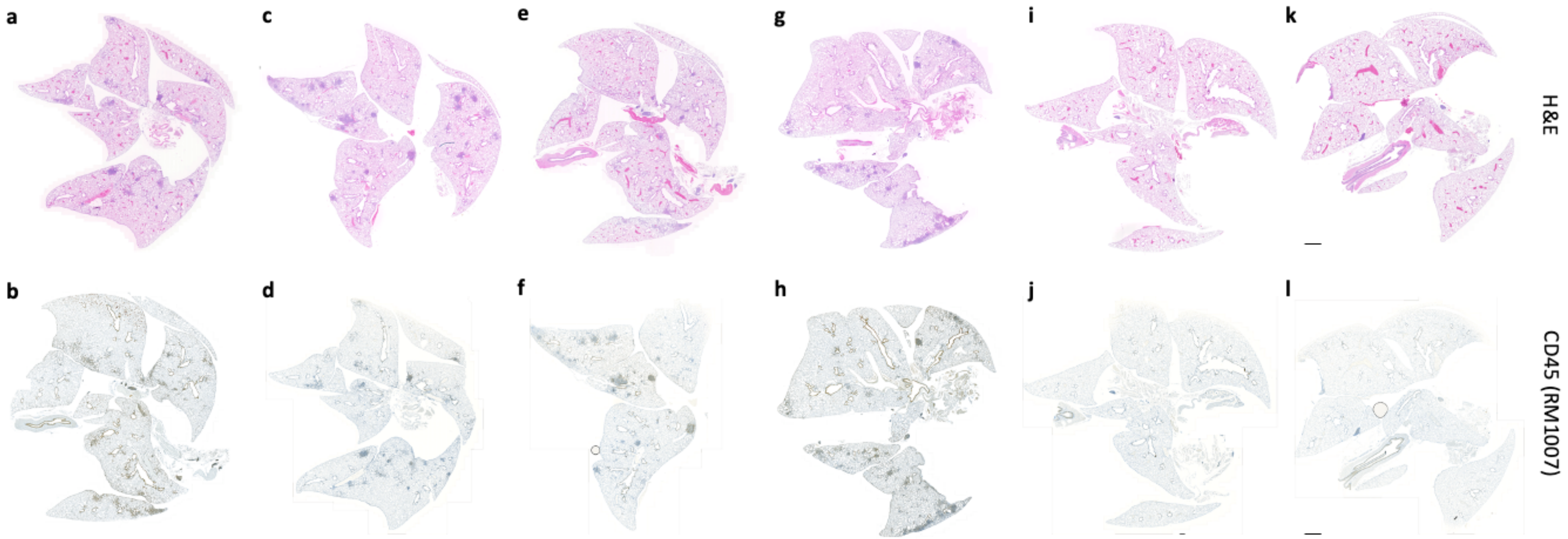

Figure S6. Hematoxylin and eosin and immunohistochemical staining of individual mouse lung tissue from three APCt-CoV2-T vaccinated mice (a,b; c,d; e,f), one APCt-Mock vaccinated mouse (g,h) and two näive mice (i,j and k,l). For CD45 staining rabbit-anti-mouse CD45 (Table S3 for details) was incubated at 1:2,000 and detected using HRP-conjugated anti rabbit (omniMap-Rabbit-HRP, Roche). Images were acquired from digital slides obtained using a Zeiss AxioScan equipped with a 20x objective. Scale bar: 1 mm.
